# The Effects of Sewage Sludge Biochar on Rhizosphere Microbial Community, Soil Quality, and *Ryegrass* and *Cosmos* Growth in Pot Culture

**DOI:** 10.3390/plants14050641

**Published:** 2025-02-20

**Authors:** Yang Yang, Hongjie Wang, Wenyi Dong, Qitian Li, Qi Han, Chaoxiang Li, Tianhao Liu, Pingyan Zhou

**Affiliations:** 1 School of Civil and Environmental Engineering, Harbin Institute of Technology Shenzhen, Shenzhen 518055, China; yang.yang@hit.edu.cn (Y.Y.); wenyidong2024@163.com (W.D.); 13719829273@163.com (Q.L.); qihan@hit.edu.cn (Q.H.); lichaoxiang2023@126.com (C.L.); tianhao.l@foxmail.com (T.L.); zpyxglzka777@163.com (P.Z.); 2 Shenzhen Key Laboratory of Water Resource Utilization and Environmental Pollution Control, Shenzhen 518055, China; 3 State Key Laboratory of Urban Water Resource and Environment, School of Environment, Harbin Institute of Technology, Harbin 150090, China; 4 Joint Laboratory of Urban High Strength Wastewater Treatment and Resources Utilization, Shenzhen 518055, China

**Keywords:** sewage sludge biochar, plant growth, soil nutrients, enzyme activities, rhizosphere soil microbial community

## Abstract

Sewage sludge biochar (SSB) is an innovative environmental material with remediation capabilities and significant potential for soil enhancement. This study aimed to accurately assess the dual regulatory effects of SSB on plant growth and soil quality. We conducted potting experiments with *ryegrass* and *cosmos* to analyze the impacts of SSB on plant growth, soil quality, and microbial communities. The partial least squares path model (PLS-PM) analysis was employed to elucidate the intrinsic relationships between SSB application and soil environmental factors, microbial communities, and plant growth. The results indicated that the application of SSB significantly enhanced the growth of *ryegrass* and *cosmos*, improved the soil quality, and increased the quantity of soil beneficial bacteria in the inter-root soil microbial communities. The addition of 9% and 3% (w w^−1^) SSB resulted in the most substantial growth of *ryegrass* and *cosmos*, with aboveground biomass increasing 68.97% and 68.12%, respectively, and root biomass increasing by 49.87% and 45.14%. PLS path analysis revealed that SSB had a significant effect on the number of bacteria, which also played an important role in soil environmental factors such as pH and conductivity. This study provides a scientific basis for the utilization of sludge resources, green agriculture, and soil improvement. Additionally, it offers technical support for optimizing the application strategy of sludge biochar.

## 1. Introduction

China’s sludge disposal and resource recovery industry started relatively later than that of other countries [1]. Despite the growing sophistication of wastewater treatment processes, a large amount of byproducts (residual sludge) are generated during wastewater treatment [2]. Coupled with high disposal costs, the safe and effective management of sludge has emerged as a pressing urban problem that demands urgent attention [3]. The traditional methods of sludge treatment and disposal, such as sanitary landfilling and incineration, not only waste the inherent resource value of sludge but also contribute to secondary environmental pollution [4]. From the perspective of long-term sustainable development, sludge contains a vast amount of recoverable resources [1]. Efficient and environmentally friendly disposal of sludge, while simultaneously harnessing these valuable components, holds great practical significance and economic value to efficiently and cleanly dispose of sludge while simultaneously utilizing these valuable components.

Pyrolysis of sludge can produce residual solid material (sewage sludge biochar (SSB)) while achieving harmless treatment of the sludge [5]. With the ongoing advancement of research by scholars, sludge pyrolysis and carbonization technology has emerged as a prominent area of study in recent years [6,7]. This technology is dedicated to developing treatment approaches for sludge with diverse properties and origins. It offers potential for nutrient recovery, energy recovery, heavy metal stabilization, and environmental conservation [8,9]. Studies show SSB, similar to other biochars prepared from waste materials (such as agricultural and forestry waste), is abundant in surface functional groups [10,11,12]. SSB has a developed porous structure with a large specific surface area, which makes it an excellent adsorbent material and soil amendment [13,14]. Mixing SSB with soil in a certain proportion enhances the soil’s capacity to retain nutrients and water [7]. Since SSB contains essential nutrients such as nitrogen, phosphorus, and potassium. Its application to soil can enhance soil chemical properties and significantly promote plant growth [15]. While biochar modifies the soil environment, it also impacts the dominant bacterial populations in the soil, thereby influencing the structure of the microbial community [16,17]. Nevertheless, it is currently unclear whether the potential application of SSB for lawn soil remediation and quality enhancement remains. Lawn soil is one of the most severely disturbed types of soil due to human activities. Soil degradation is significant due to reduced permeability caused by construction disturbances and compaction [18,19]. In landscaping, lawns play a crucial role in improving the urban ecology and beautifying the living environment. In developed countries, peat and municipal waste compost are commonly utilized to improve the quality of landscaping soil [20]. However, China has limited peat resources, and garbage compost is insufficient to meet the requirements for landscaping soil quality [20]. Therefore, developing innovative methods to enhance the quality of landscaping soil holds significant value and meaning.

In this study, SSB was applied to lawn soil, and two commonly used lawn plants (*ryegrass* and *cosmos*) were selected for pot experiments. The objectives of this study were (1) to investigate the effects of SSB on the growth, chlorophyll content, and enzyme activity of *ryegrass* and *cosmos* plants; (2) to explore the response characteristics of SSB on soil nutrients, enzyme activity, and microbial diversity in the soil where *ryegrass* and *cosmos* were planted; (3) to ascertain the interaction mechanisms between soil environmental variables and soil microbial components; and (4) to determine the appropriate application rate of SSB in lawn soil.

## 2. Results

### 2.1. Effect of Sewage Sludge Biochar on Ryegrass and Rosmos Growth

**Figure 1:**
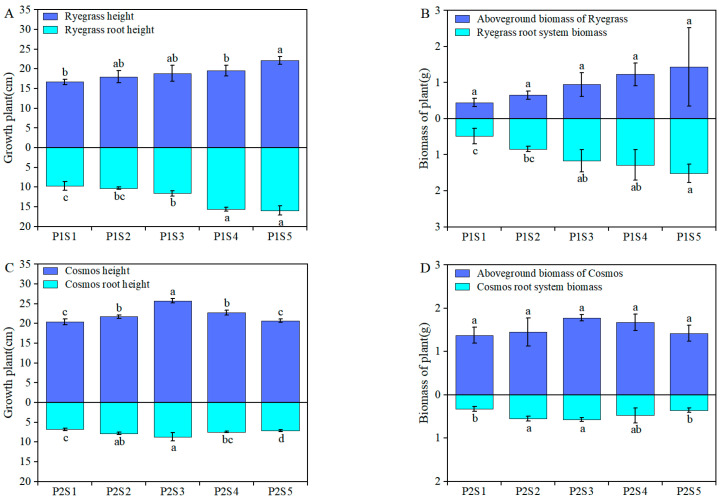
The impact of adding SSB on plant growth and biomass. Plant and root height of both *ryegrass* (**A**) and *cosmos* (**C**), as well as the aboveground and root system biomass of *ryegrass* (**B**) and *cosmos* (**D**). According to Tukey’s test, different letters are used to indicate significant differences (*p* < 0.05). Note: P1S1, *ryegrass* + 0% SSB, w w^−1^; P1S2, *ryegrass* + 1% SSB, w w^−1^; P1S3, *ryegrass* + 3% SSB, w w^−1^; P1S4, *ryegrass* + 6% SSB, w w^−1^; P1S5, *ryegrass* + 9% SSB, w w^−1^; P2S1, *cosmos* + 0% SSB, w w^−1^; P2S2, *cosmos* + 1% SSB, w w^−1^; P2S3, *cosmos* + 3% SSB, w w^−1^; P2S4, *cosmos* + 6% SSB, w w^−1^; P2S5, *cosmos* + 9% SSB, w w^−1^.

After completing the pot experiment (40 days post-planting), Figure 1 illustrates the growth of *ryegrass* and *cosmos* under various sewage sludge biochar (SSB) applications. The SSB treatment demonstrated a more significant positive effect on the aboveground and root biomass of *ryegrass* compared with *cosmos*, as shown in Figure 1A,C. Compared with the control treatments (P1S1 and P2S1), the application of different SSB resulted in a significant increase in the aboveground and root biomass of *ryegrass* and *cosmos*, with increases ranging from 3.17% to 68.97% and 9.05% to 68.12%, respectively (Figure 1B,D). This indicates that the addition of SSB can enhance the biomass of *ryegrass* and *cosmos*, which is beneficial to plant growth. The highest increase in biomass was observed in the P1S5 and P2S3 treatments.

### 2.2. Effect of Sewage Sludge Biochar on Chlorophyll Content and Antioxidant Enzymne Activity

**Figure 2:**
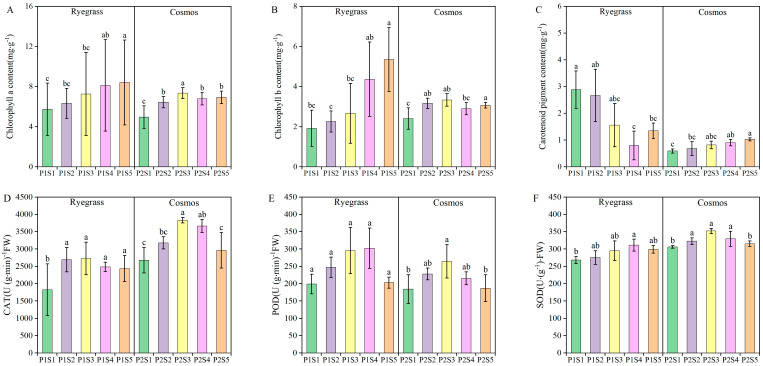
The effects of SSB application on plant photosynthetic pigments, including chlorophyll a (**A**), chlorophyll b (**B**) and carotenoid pigments (**C**), as well as antioxidant enzyme activities CAT (**D**), POD (**E**) and SOD (**F**) contents. According to Tukey’s test, different letters are used to indicate significant differences (*p* < 0.05). Note: Same as in Figure 1.

Compared with the control treatments (P1S1 and P2S1), the application of SSB significantly increased the contents of chlorophyll a, chlorophyll b, and carotenoid pigments in *ryegrass* and *cosmos* by 10.30% to 48.41%, 17.91% to 178.64%, and 8.19% to 57.58%, respectively (*p* < 0.05) (Figure 2A–C). With the increasing applications of SSB, the chlorophyll content in *ryegrass* exhibited an upward trend, while in *cosmos*, it initially rose and subsequently declined. This suggested that the application of SSB could enhance the chlorophyll content, thereby facilitating photosynthesis and growth, with the most significant increases observed in the P1S5 and P2S3 treatments. The application of SSB enhanced the activities of the antioxidant enzymes CAT, POD, and SOD in both *ryegrass* and *cosmos*, exhibiting a general trend of first increasing and then decreasing (Figure 2D–F). Compared with the control treatments (P1S1 and P2S1), the activities of the antioxidant enzymes CAT, POD, and SOD increased by 18.56% to 49.61%, 1.37% to 52.09%, and 2.99% to 16.02%. Overall, the most significant increase in antioxidant enzyme activity was observed in the P1S3 and P2S3 treatments.

### 2.3. Effect of Sewage Sludge Biochar on Soil Quality

**Figure 3:**
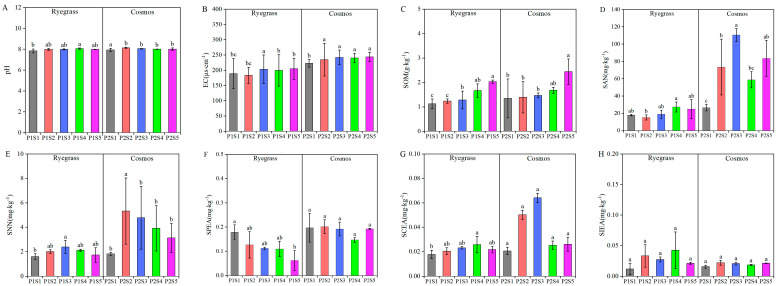
Effects of SSB application on soil physicochemical properties, including pH (**A**), EC (**B**), SOM (**C**), SAN (**D**) and SNN (**E**), as well as enzyme activities such as SPEA (**F**), SCEA (**G**) and SIEA (**H**). According to Tukey’s test, different letters are used to indicate significant differences (*p* < 0.05). Note: EC—soil electric conductivity; SOM—soil organic matter; SAN—soil N-NH_4_; SNN—soil N-NO_3_; SPEA—soil phosphatase activity; SCEA—soil cellulose enzyme activity; SIEA—soil sucrase activity.

The results of the soil physicochemical properties and enzyme activity from *ryegrass* and *cosmos* are presented in Figure 3. When SSB was applied, the soil physicochemical properties (pH, EC, SOM, SAN, and SNN) of both *ryegrass* and *cosmos* increased compared with the control treatments (P1S1 and P2S1). Among them, pH, EC, SOM, SAN, and SNN increased by 0.01% to 2.85%, 2.3% to 9.54%, 3.37% to 81.21%, 1.40% to 315.81%, and 8.56% to 194.34%, respectively. Furthermore, with the application of SSB, there was minimal change in pH, while EC and SOM exhibited an increasing trend. SAN and SNN initially increased and then decreased (Figure 3A–D). The soil enzyme activity displayed varying responses depending on the amount of SSB applied. Except for SPEA, which showed a decreasing trend, both SCEA and SIEA exhibited a trend of first increasing and then decreasing. This suggested that applying SSB could influence soil physicochemical properties and enzyme activity.

### 2.4. Effect of Sewage Sludge Biochar on Rhizosphere Micorbial Coomunity Structure Under Ryegrass and Cosmos

**Figure 4:**
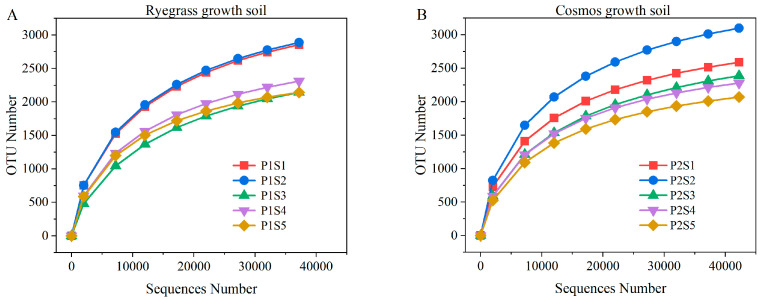
Bacterial rarefaction curves for *ryegrass* (**A**) and *cosmos* (**B**) growth soil under different treatments.

A total of 413,019 effective bacterial gene sequences were obtained from all soil samples through high-throughput sequencing. The effective tags from all samples were clustered into OTUs (operational taxonomic units) at 98% identity, with the bacterial sequence aggregation level ranging from 2071 to 3099 OTUs. The results of the soil bacterial rarefaction curves for *ryegrass* and *cosmos* are presented in Figure 4. The rarefaction curves for bacterial taxa revealed a distinct trajectory approaching saturation, suggesting a significant proportion of microbial diversity information during plant growth (Figure 4). Under the influence of SSB, the bacterial diversity in soil planted with *ryegrass* and *cosmos* planted soil decreased with the increase in the application amount. However, the Chao1 and ACE indices initially increased before subsequently declining. The lower Shannon index and the higher Simpson index indicated reduced diversity within bacterial communities. Compared with the control treatments (P1S1 and P2S1), the application of SSB to the soil supporting *ryegrass* and *cosmos* resulted in a decrease in the bacterial Shannon index and an increase in the Simpson index (Table 1). This indicated that the use of SSB could decrease the diversity and evenness of bacterial communities in the soil, with P1S3 and P2S5 showing the most significant effects. Interestingly, the number of beneficial bacteria in the rhizosphere soil increased with the higher application rates of SSB. The Good’s coverage index for all treatments was greater than 0.984, suggesting that the sequencing depth was adequate to accurately represent the true bacterial composition in the soil under each treatment.

**Figure 5:**
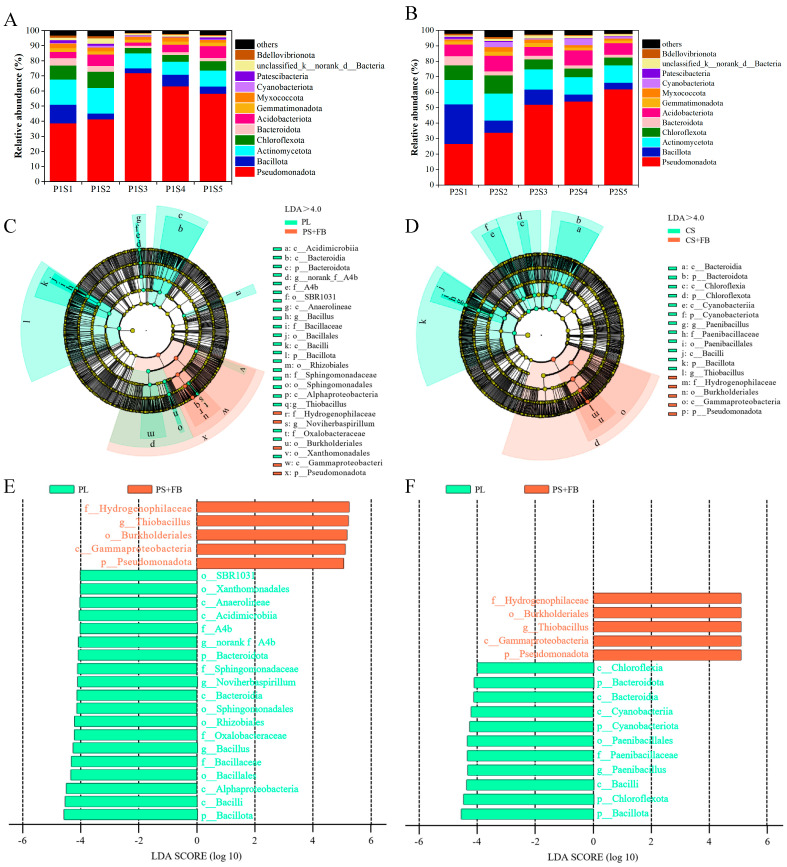
The relative abundance of bacteria microorganisms in the growth soil of *ryegrass* (**A**) and *cosmos* (**B**) is presented at the phylum level (%). The linear discriminant analysis effect size (LEfSe) delineates disparities in *ryegrass* (**C**) and *cosmos* (**D**) communities under diverse treatments in the rhizosphere soil. The linear discriminant analysis (LDA) for the taxonomic groups of *ryegrass* (**E**) and *cosmos* (**F**) identifies the most distinguishing groups among the different treatments. The concentric circles symbolize the seven taxonomic levels, ranging from phylum to genus, with the size of the circles indicating the relative abundance of bacteria.

**Figure 6:**
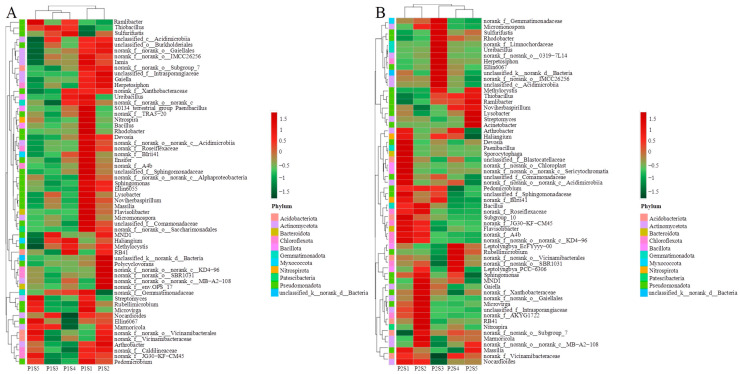
Distribution heat map of the top 60 bacteria of *ryegrass* (**A**) and *cosmos* (**B**) growth soil at the genus level in rhizosphere soil.

The overall taxonomic classification of eligible OTUs identified 47 distinct phyla of bacteria. *Pseudomonadota*, *Bacillota*, *Actinomycetota*, and *Chloroflexota* constituted the predominant bacterial phyla, accounting for 71.18% to 88.53% of the phylum-level composition (Figure 5A,B). Compared with the control treatments (P1S1 and P2S1), the abundance of *Pseudomonadota* in the *cosmos* growth soil increased, while the levels of *Bacillota*, *Actinomycetota*, and *Chloroflexota* decreased (Figure 5A). In the *ryegrass* growth soil, *Pseudomonadota* and *Actinomycetota* also increased, whereas *Bacillota* and *Chloroflexota* decreased (Figure 5B). The relative abundance of the top 60 bacterial genera revealed that 23 of them belonged to the phylum *Pseudomonadota*. Cluster analysis indicated that the number of different types of bacteria showed similar trends between P1S3 and P1S4, P2S5, and P2S4 treatments in bacterial communities. The genera with the highest relative abundance of genus were *Thiobacillus*, *Bacillus*, and *Lysinibacillus* (Figure 6).

Treatments were categorized into two groups: soil without SSB, which included PL (P1S1) and CS (P2S1), and soil with different content of SSB, PS + FB (P1S2, P1S3, P1S4, and P1S5) and CS + FB (P2S2, P2S3, P2S4, and P2S5). The impacts of SSB application on the physicochemical properties of soil and the changes in *ryegrass* and *cosmos* were examined. The linear discriminant analysis effect size (LEfSe) (Figure 5C,D) and linear discriminant analysis (LDA) (Figure 5E,F) provided detailed insights into the richness and structure of soil bacterial communities. Within the bacterial communities, the family *Hydrogenophilaceae* was found be more abundant in the PS + FB and CS + FB treatment groups, whereas the order *SBR1031* and the class *Chloroflexota* were more prevalent in the PL and CS treatment groups.

**Figure 7:**
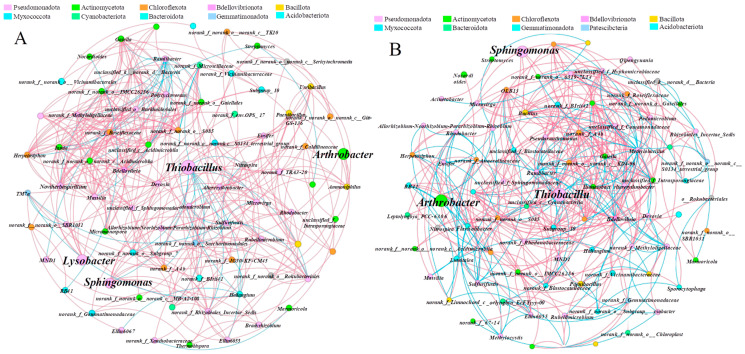
The co-occurrence network analysis at the genus level for *ryegrass* (**A**) and *cosmos* (**B**) growth soil provides a visual representation of microbial interactions with a relative abundance > 1 %. The network selectively illustrates robust (|*r*| > 0.6) and statistically significant (*p* < 0.01) relationships. Nodes in the network are color-coded by phylum, while edges represent the correlation between two connected nodes, with magenta indicating a positive correlation and cyan indicating a negative correlation.

Through co-occurrence network analysis, we conducted an in-depth exploration of potential key microbial groups in soil. We constructed a co-occurrence network of soil bacterial groups of *ryegrass* and *cosmos*, as applied by SSB, at the genus level (Figure 7A,B). For *ryegrass*, the key bacterial genera identified in the soil network included *Thiobacillus*, *Sphingomonas*, *Arthrobacter*, and *Lysobacter*, with the highest connectivity centrality reaching 53. The bacteria associated with *Thiobacillus* mainly belonged to *Pseudomonadota*, *Chloroflexota*, *Actinomycetota*, etc. (Figure 7A). Among these, 34 bacterial genera exhibited a significant negative correlation with *Thiobacillus*, while 4 bacterial genera demonstrated a significant positive correlation. For *cosmos*, *Thiobacillus*, *Arthrobacter*, and *Sphingomonas* were identified as the key bacterial genera in the soil network. Additionally, 51 bacterial genera exhibited a significant negative correlation with *Thiobacillus*, while 12 bacterial genera showed significant positive correlations. The co-occurrence network clustering coefficients of soil bacterial communities associated with *ryegrass* and *cosmos* exceeded 0.54, indicating strong cooperation among microorganisms. According to the network topological properties of the microbial co-occurrence networks (Table 2), the number of nodes and edges in the soil of *cosmos* was higher than that of *ryegrass*, and it also exhibited a higher average degree and betweenness centralization.

## 3. Discussion

### 3.1. Effect of Sewage Sludge Biochar Application on Plant Growth, Chlorophyll Content, and Enzyme Activity

Plant height and biomass are important traits of plants that can directly influence the nutrient utilization efficiency of plants. The morphology of plant roots serves as an indicator of soil quality, reflecting both its physical and chemical characteristics [21,22]. Appropriate plant height and biomass can improve the utilization efficiency of photosynthesis, nutrients, and water [23]. Prior research has showed that the sewage sludge biochar (SSB) was rich in nitrogen, phosphorus, potassium, and micronutrient contents, which could improve wheat growth [24,25]. As expected (Figure 1), compared with the control treatments, the addition of SSB to the soil not only promoted plant and root growth but also increased both plant and root biomass, consistent with other studies [26,27]. The addition of 9% and 3% (w w^−1^) SSB significantly increased the height and biomass of *ryegrass* and *cosmos* plants (Figure 1). During plant growth, chlorophyll, a vital pigment in photosynthesis, influences enzyme activity and facilitates the conversion of carbon dioxide and water into organic matter, thereby affecting plant growth and metabolism [28,29]. The higher chlorophyll content correlates with an increased photosynthetic rate in leaves, promoting plant growth [30]. This study found that SSB increased the chlorophyll content of *ryegrass* and *cosmos* to varying degrees (Figure 2). This enhancement may have been due to the application of SSB increasing soil nutrients, while the increased specific surface area of the SSB–soil mixture enhanced the soil’s nutrient adsorption capacity, reducing nutrient loss and allowing plants to better absorb essential elements from the soil [24,31]. Antioxidant enzymes (CAT, POD, and SOD) are present in plants. They play a crucial role in regulating the levels of reactive oxygen species (ROS) during normal physiological metabolism [32]. These enzymes can eliminate detrimental ROS and maintain the stability and intactness of cellular membranes [33]. In this study, as the amount of SSB application increased, the activities of CAT, POD, and SOD in *ryegrass* and *cosmos* leaves initially increased and then decreased. The antioxidant enzymes activity in P1S3 and P2S3 treatments was the highest. This indicated that the application of SSB could enhance the activity of antioxidant enzymes in plants [34,35].

### 3.2. Impact of Sewage Sludge Biochar on Soil Quality

The improvement in soil quality conditions enhances the growth environment for plants and strengthens their resistance to stress [36,37], enabling them to better cope with adverse conditions such as drought, pests, and diseases and thus maintaining stable growth [38]. Soil physicochemical properties such as pH, EC, SOM, SAN, and SNN play an essential role in soil fertility, as well as plant growth and development. In the present study, the application of SSB significantly improved the soil pH and EC compared with the control treatments (P1S1 and P2S1) (Figure 3A–E). The soil pH increased after biochar was applied, primarily due to the main component of ash in biochar produced from different materials during calcination being carbonate, which caused biochar to have a higher pH value [39,40]. The soil OM, SAN, and SNN were significantly higher than those observed in the control treatments. These observed increases could be attributed to the demonstrated effects of SSB application, which effectively modulated the soil pore distribution, enhanced the soil structural integrity, and promoted more efficient nutrient uptake during various plant growth stages [41,42]. Overall, we observed that SSB application improved the soil’s ability to stabilize nutrients.

Soil enzymes play a crucial role in soil biochemical processes, which affect the transformation of organic matter and biogeochemical cycles, serving as significant indicators of soil fertility [43,44]. In the present study, the results showed that the addition of SSB significantly increased SCEA and SIEA (Figure 3G,H). The fluctuating activity of soil enzymes impacts nutrient cycling, respiration, N_2_O emissions, and the breakdown of organic matter [45]. Furthermore, the increase in soil enzymatic activities may have been induced by increased soil nutrients, which provide organic substrates for soil enzymes [46]. In the current study, the higher soil cellulose enzyme activity upon biochar addition supported the findings of the present study [47]. Prior research has shown that subject to the environment, the biochar–enzyme interactions can enhance [48], decrease [49], or have no influence on enzyme activity. Similarly, the results of our study indicated a decrease in SPEA under SSB application as compared with the control treatments (P1S1) (Figure 3F).

**Figure 8:**
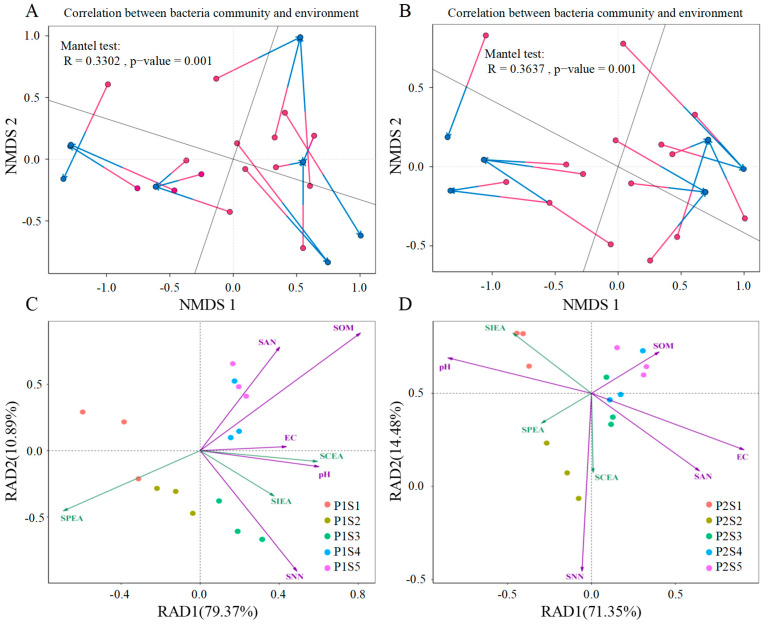
The redundancy analysis was conducted on the counts of environmental factors for *ryegrass* (**A**) and *cosmos* (**B**) growth soil. A Procrustes analysis was performed to determine the correlation between environmental factors and all microbial communities, based on the non-metric multidimensional scaling (NMDS) results (Bray–Curtis) for the abundance of environmental factors and all OTUs in *ryegrass* (**C**) and *cosmos* (**D**) growth soil communities within the inter-rhizosphere soil samples (999 permutations). The arrows in the analysis indicate the magnitude and direction of environmental factors associated with the structure of the bacterial community. The *p*-values represent the results of significance tests for differences between environmental factors and soil microbial communities.

Procrustes analysis was utilized to assess the correlation between rhizosphere soil environmental factors (SAN, SOM, pH, SNN, EC, SPEA, SIEA, and SCEA) and the whole microbial community of the two plant species (Figure 8). The results showed that the overall changes in environmental factors significantly impacted the bacterial community in both *ryegrass* (Mantel test, *R* = 0.3302, *p* ≤ 0.001) and *cosmos* (Mantel test, *R* = 0.33637, *p* ≤ 0.001), and the changes in environmental factors had a more pronounced influence on the bacterial community structure of *cosmos* (Figure 6A,B). Additionally, redundancy analysis was conducted to determine the correlation between rhizosphere bacteria and rhizosphere soil environmental factors in both *ryegrass* and *cosmos* (Figure 8C,D). The significance of the correlation between each environmental parameter and the microbial community is presented in Table 3. Soil nutrient status was a crucial factor influencing the bacterial community structure, including SAN, SOM, pH, and SNN, with SNN being the most critical. Additionally, the structure of soil bacterial communities was also affected by soil enzyme activity (SPEA, SIEA, and SCEA), with SPEA and SIEA identified as the most significant factors.

**Figure 9:**
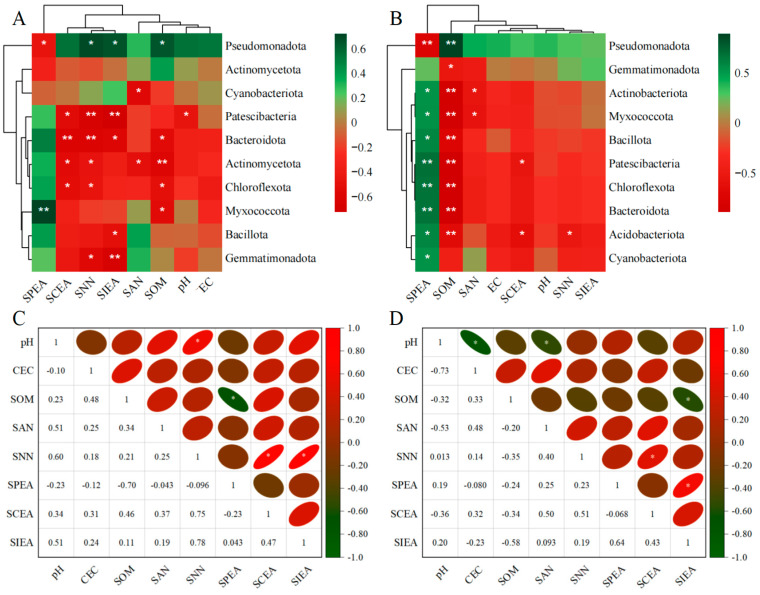
Spearman correlation analysis between rhizosphere soil environmental factors and the top 10 bacterial phyla for *ryegrass* (**A**) and *cosmos* (**B**) growth soil. Heat maps of the rhizosphere soil environmental factors for *ryegrass* (**C**) and *cosmos* (**D**) growth soils are presented. Significance levels are denoted as ** for *p* < 0.01 and * for *p* < 0.05.

The clustering analysis of rhizosphere soil environmental factors and the abundance of the top 10 bacterial phyla in the rhizosphere soils of *ryegrass* and *cosmos* is illustrated in Figure 9A,B. The results indicate that soil nutrients (SCEA, SOM, and SNN) exhibit a high degree of consistency in their influence on changes at the bacterial phylum level, positively correlating with most dominant phyla, except for *Pseudomonadota* and *Actinomycetota*. Meanwhile, *Pseudomonadota* displays a negative correlation with all rhizosphere soil environmental factors, except for SPEA. The soil microbial community was significantly influenced by SOM. Through Spearman correlation analysis, additional correlation studies were conducted on the environmental factors of rhizosphere soil. It was observed that in the rhizosphere soil of *ryegrass*, there was a significant positive correlation between pH and SNN and a significant positive correlation between SNN, SCEA, and SIEA. Additionally, a significant negative correlation was found between SOM and SPEP (Figure 9C). Among the environmental factors of rhizosphere soil in *cosmos*, SNN showed a significant positive correlation with SCEA, while SPEA exhibited a significant positive correlation with SIEA. Additionally, pH demonstrated a significant negative correlation with EC, and SAN and SOM were significantly negatively correlated with SIEA (Figure 9D).

### 3.3. Effect of Sewage Sludge Biochar on Microbial Community and Structural Diversity

Soil microorganisms are an essential component of soil, responsible for decomposing plant residues into humus [50]. They serve as a reserve pool for plant-available nutrients, capable of storing and releasing these nutrients to meet the growth demands of plants [51]. Consequently, soil microorganisms play a crucial role in soil fertility and plant nutrition [52]. The transformation and absorption of nutrients between plants and soil are closely related to the quantity of microorganisms and the structure of their communities [53,54]. Research findings indicate that incorporating biochar leads to an increase in the abundance of bacteria and fungi [55]. Similarly, the present study found that under the influence of SSB treatments, both the Chao1 and Shannon indices for bacterial communities were lower than those in the control treatments, exhibiting a decreasing trend with increasing SSB dosage (Table 1). SSB facilitated the growth and survival of beneficial soil bacteria, a conclusion supported by the results from the co-occurrence network analysis (Figure 7). The abundance of *Pseudomonadota* in the rhizosphere soil increased with SSB treatments. In terms of community composition and relative abundance, *Pseudomonadota* composed the largest proportion of the soil community, aligning with previous findings [56,57]. This methodology increased the population of beneficial soil bacteria, fostering the development of a stronger and more diverse microecological environment within the rhizosphere. Within the bacterial community, the addition of SSB to the soil resulted in an increase in the proportion of *Pseudomonadota*, which resulted in the relative abundance of *Bacillota*.

The LEFSe analysis indicated that *Pseudomonadota* were the significantly dominant bacterial biomarkers under SSB treatment. *Pseudomonadota* are capable of fixing nitrogen and participating in processes such as sulfur oxidation and methane oxidation, which promote plant growth and significantly impact ecosystem [58]. *Bacillota* play a crucial role in the decomposition of organic matter and nitrogen compounds in the soil, contributing to the release of available nutrients [59]. In this experiment, *Pseudomonadota* alleviated the biological stress caused by soil pathogenic bacteria and promoted plant growth. This was evidenced by the increase in the relative abundance of *Pseudomonadota* as the amount of applied SSB increased. Furthermore, after the application of SSB, *Thiobacillus* (genus) emerged as a significantly dominant bacterium in the treated group, belonging to the *Pseudomonadota* phylum. SSB may have provided a niche that facilitated the colonization of *Pseudomonadota*, thereby reducing the space and resources available for *Bacillota*. This niche competition may have contributed to the decline of *Bacillota* populations. Research has shown that *Pseudomonadota* can interact positively with plants and produce extracellular *polysaccharides*, which enhance the proliferation of bacterial communities [58].

The results of the co-occurrence network analysis indicated that *Thiobacillus* exhibited greater biological connections in the treatment, significantly enhancing its role in the soil bacterial community. The application of SSB increased the organic matter content in the rhizosphere soil (Figure 3C). Both *Thiobacillus* and *Sphingomonas* possess good organic matter decomposition capabilities, positively contributing to the quality of rhizosphere soil and the abundance of bacterial communities [60]. Consequently, the application of SSB modified the soil environment, which in turn affected the species composition, abundance, activity, and structure of microorganisms.

### 3.4. Comprehensive Evaluation of Soil Quality Index

**Figure 10:**
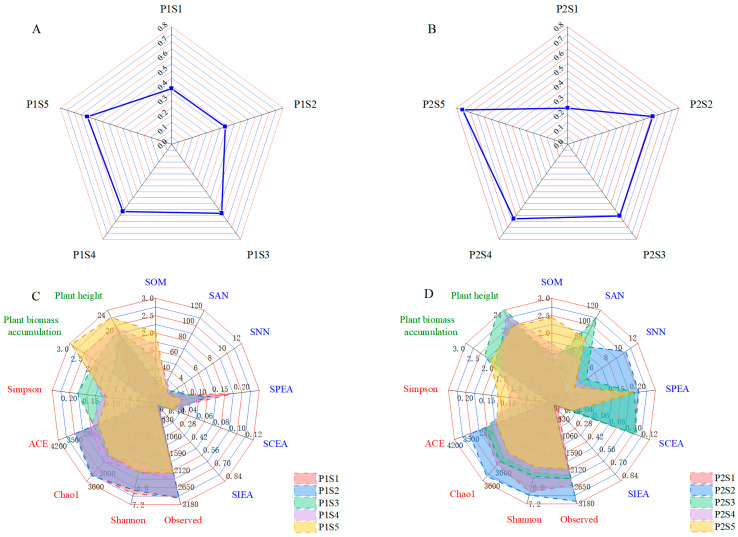
Radar charts depict the SQI at *ryegrass* (**A**) and *cosmos* (**B**) growth soil. The radar plots demonstrate the association between rhizosphere soil health and plant development in *ryegrass* (**C**) and *cosmos* (**D**) growth soil. Soil parameters are depicted in blue, microbial parameters are highlighted in red, and plant parameters are represented in green.

The fuzzy comprehensive evaluation method was utilized to assess the membership functions and weights of various indicators pertaining to the soil properties of *ryegrass* and *cosmos* (Table 4). Analysis of the weights assigned to each parameter revealed the following hierarchy of the impact on soil quality: for *ryegrass*, pH (0.263) > SIEA (0.154) > EC (0.149) > SAN (0.136) > SPEA (0.125) > SOM (0.077) > SCEA (0.062) > SNN (0.034), and for *cosmos*, pH (0.254) > SOM (0.199) > EC (0.180) > SNN (0.121) > SAN (0.103) > SPEA (0.074) > SCEA (0.042) > SIEA (0.027). The radar chart was utilized to represent the comprehensive soil quality with different SSBs (Figure 10A,B). Compared with the control treatments (P1S1 and P2S1), the application of SSB significantly improved the soil quality for both *ryegrass* and *cosmos*, with improvements generally increasing alongside the application rate. Among the *ryegrass* planting soils, P1S5 exhibited the highest soil quality (level III > 0.55), while P1S3 and P1S4 demonstrated comparable SQI values (level III > 0.55).

For *cosmos* planting soils, P2S5 showed the best soil quality (level II > 0.7), with P2S2 and P2S3 having similar SQI values. Furthermore, the radar chart was used to evaluate plant growth and rhizosphere soil health conditions following different treatments (Figure 10C,D). The areas under the curves for each treatment surpassed those of the control treatments (P1S1 and P2S1). The largest area observed for *ryegrass* was P1S4, followed by the P1S5 and P1S3 treatments. For *cosmos*, the largest area was noted in P2S3, followed by the P2S2 and P1S4 treatments.

The SQI (soil quality index) is a key parameter for evaluating soil quality [61]. This study found that in *ryegrass* and *cosmos* soils, pH and EC had the most significant influence on the SQI, followed by soil nutrients and enzyme activity. The presence of SSB promoted nutrient cycling and enhanced the structural stability of the soil, thereby enhancing the growth of *ryegrass* and *cosmos* (Figure 10). Compared with the control, SSB significantly improved the SQI value. After applying SSB, the soil structure was enhanced, and the soil’s nutrient retention capacity increased [62], leading to an overall improvement in soil quality. In the control treatment, the soil was classified as V (SQI < 0.4). As the application rate of SSB increased, the SQI value showed a significant rise. It was hypothesized that SSB, by improving soil structure, allowed nutrients to be better fixed in plant roots, allowing plants to fully absorb these nutrients and thereby promoting plant growth [19].

### 3.5. Cascade Relationship Between Sewage Sludge Biochar, Soil Environmental Factors, Microbial Communities, and Plant Growth

**Figure 11:**
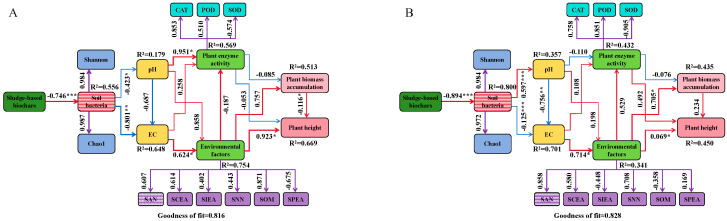
Structural equation model of the physiological growth influence mechanisms for *ryegrass* (**A**) and *cosmos* (**B**). In the outer model, the number on the purple arrow indicates the external model load. In the inner model, red and blue arrows represent positive and negative causal relationships, respectively. The numbers on the arrows denote significant standardized path coefficients. R^2^ represents the variance of the dependent variable explained by the model. Significance levels are indicated as follows: * for *p* < 0.05, ** for *p* < 0.01, and *** for *p* < 0.001.

Partial least squares path modeling (PLS-PM) clarified the primary pathways through which soil environmental factors and microbial communities influenced by SSB application affected plant growth, as well as the overall impact of each variable on plant biomass accumulation and height (Figure 11A,B). The goodness of fit (GoF) of the model was 0.816 and 0.828, indicating that the model demonstrated a good fit and successfully passed the overall model fit test. The results indicated that the application of SSB significantly impacted the soil bacterial quantity (measured by Chao1 and Shannon indices) in *ryegrass* (path coefficient = 0.987 and 0.984, *p* < 0.05) and *cosmos* (path coefficient = 0.972 and 0.9884, *p* < 0.005). Soil bacteria significantly affected both pH and EC, with pH negatively impacting EC. Additionally, EC significantly influenced environmental factors. These environmental factors had a significant positive impact on plant biomass accumulation and height. Therefore, it could be concluded that SSB could alleviate pH and EC levels, enhance plant enzyme and environmental factors by increasing the soil bacterial population, and then indirectly increase *ryegrass* and *cosmos* biomass accumulation and height.

## 4. Materials and Methods

### 4.1. Materials

The test sludge was obtained from the thickening tank of the Nanshan Water Purification Plant in Shenzhen, Guangdong Province, China, and was sieved using a 24-mesh screen to obtain the original sludge for testing. The experiment was conducted from March to April 2024 in Shenzhen City, Guangdong Province, China (22°32′29″ N, 114°03′35″ E). The physicochemical properties of the planting soil were as follows: soil texture composed of clay (18.22%), silt (45.98%), and sand (35.80%); pH of 8.22 (1:1 w v^−1^); organic matter, 20.98 (mg kg^−1^); ammonium nitrogen, 20.28 (mg kg^−1^); and nitrate nitrogen, 5.83 (mg kg^−1^). The original sludge had a pH of 6.7 ± 0.3, a total suspended solids (TSS) content of 32 ± 5.0 g L^−1^, a volatile solids content (VSS) of 17.6 ± 3.3 g L^−1^, an organic matter content of 54.9 ± 2.3% (w w^−1^), and a moisture content of 96.8 ± 0.4%. In the study, *ryegrass* and *cosmos* seeds were sourced from the same batch of seed distributors (Jiangsu Chengying Landscaping Co., Ltd.). The purchased seeds were surface-disinfected with a 2% sodium hypochlorite solution for 30 min, followed by rinsing this with sterilized distilled water three times to remove any residual solvent from the surface.

### 4.2. Preparation of Sewage Sludge Biochar

Fenton conditioning sludge (the product of raw sludge conditioned with 0.8 g g^−1^ TSS of ferrous sulfate heptahydrate and 0.8 g g^−1^ TSS of 30% hydrogen peroxide) was used as the preparation material for sludge biochar [63]. Following dehydration using a filter press, the sludge was placed in a blast drying oven and dried at 105 °C until constant weight. The dried sample was then placed in a vacuum tube furnace and pyrolyzed under an N_2_ atmosphere (flow rate of 50 mL min^−1^) at 1000 °C (heating rate of 5 °C min^−1^) for 2 h. The resulting pyrolyzed biochar was ground and sieved through a 100-mesh screen to produce the experimental sewage sludge biochar (SSB). The fundamental composition of the SSB was measured by the ICP-MS 7700x (Agilent Technologies, California, USA) with 0.2 g of SSB samples, as presented in Table 5.

### 4.3. Experimental Design and Sample Collection

Two commonly used municipal greening plants were selected: *ryegrass* (P1) and *cosmos* (P2). Five application gradients were set for SSB: 0%, w w^−1^ (S1); 1%, w w^−1^ (S2); 3%, w w^−1^ (S3); 6%, w w^−1^ (S4); and 9%, w w^−1^ (S5), based on previously reported studies [16]. Ten treatments were determined for the pot experiment: (1) *ryegrass* + 0% w w^−1^ SSB (P1S1), (2) *ryegrass* + 1% w w^−1^ SSB (P1S2), (3) *ryegrass* + 3% w w^−1^ SSB (P1S3), (4) *ryegrass* + 6% w w^−1^ SSB (P1S4), (5) *ryegrass* + 9% w w^−1^ SSB (P1S5), (6) *cosmos* + 0% w w^−1^ SSB (P2S1), (7) *cosmos* + 1% w w^−1^ SSB (P2S2), (8) *cosmos* + 3% w w^−1^ SSB (P2S3), (9) *cosmos* + 6% w w^−1^ SSB (P2S4), and (10) *cosmos* + 9% w w^−1^ SSB (P2S5). Each treatment was replicated three times. The SSB was mixed evenly with the planting soil, with a total of 500 g of mixed soil per bonsai. A quantity of 30 *ryegrass* and 15 *cosmos* seeds were planted in each pot for a 40-day outdoor pot experiment. The soil moisture content was maintained at approximately 70% of field capacity. Plant and soil samples were collected on the final day of the plant growth experiment. After collecting the intact plants, the plant height and root length were measured on site. A portion of the fresh plant and soil samples was stored in a refrigerator (4 °C) for later measurement of plant chlorophyll, plant enzyme activity, and soil enzyme activity. Another portion was stored in a low-temperature freezer (−80 °C) for subsequent analysis of soil microbial communities. Last, a portion of the soil was air-dried and passed through a 2 mm sieve for later measurement of soil physicochemical indicators.

### 4.4. Measurement of Plant and Soil Samples

A pH-automated analyzer PB-10 (Sartorius companies, Gogentin, Germany) was used to measure the pH of air-dried soil at a 1:2.5 (m v^−1^) soil-to-water ratio [64]. The electrical conductivity (EC) of the soil was determined using a FE-30 conductivity meter (Mettler Toledo Technology (China) Co., Ltd., Shanghai, China) with soil samples at a soil-to-water ratio of 1:5 (m v^−1^) [65]. The soil organic matter (SOM) was measured by the TOC analyzer L-CPN (Shimadzu Corporation, Kyoto, Japan) with 0.2 g of air-dried soil samples [66]. The contents of chlorophyll a, chlorophyll b, and carotenoid pigment in the plant leaves were extracted using acetone and subsequently measured using a UV-2600 visible spectrophotometer (Shimadzu Corporation, Kyoto, Japan) [67]. The nitroblue tetrazolium photochemical reduction method was used to quantify superoxide dismutase (SOD). The guaiacol method was used to quantify peroxidase (POD). The ammonium molybdate colorimetric method was used to quantify catalase (CAT) [68]. Phenylphosphate disodium colorimetry was used to quantify soil phosphatase activity (SPEA), and 3,5-dinitrosalicylic acid colorimetry was used to quantify soil cellulose enzyme activity (SCEA) and soil sucrase activity (SIEA) [68]. Soil ammonium nitrogen (SAN) and soil nitrate nitrogen (SNN) were measured with a UV-2600 visible spectrophotometer (Shimadzu Corporation, Kyoto, Japan) [69]. The height and root length of plants were gauged with a ruler, while their weight was determined using a balance.

### 4.5. Comprehensive Evaluation

To quantify the impact of SSB on soil quality, eight indicators including pH, EC, SOM, SAN, SNN, SPEA, SCEA, and SIEA were selected to calculate the soil quality index (SQI) using the total data scaling (TDS) method [70]. A comprehensive evaluation of these data using the SQI calculation was conducted. The selected indicators were classified. “The less, the better” principle was applied to pH and EC in Equation (1). The remaining indicators followed “the more, the better” principle as used in Equation (2) [71]. The determination of each index’s membership degree and weight was based on the rotating component load, alongside the contribution rate of the principal component variance and the cumulative contribution rate derived from the respective index data.

(1) $$YM=\frac{Y_{i}-M_{i}}{M_{a}-M_{i}}$$

(2) $$XN=\frac{M_{a}-Y_{i}}{M_{a}-M_{i}}$$

where *YM* and *XN* are the membership score, *Y_i_* is the measured value of the soil environmental index, *M_i_* is the minimum value of the soil index, and *M_a_* is the maximum value of the soil index.

The SQI calculation formula is presented in Equation (3) [72]:

(3) $$SQI=\sum_{i=1}^{m}Q_{i}S_{i}$$

where *Q_i_* represents the score of the index, m denotes the number of soil indicators in the TDS method, and *S_i_* is the weight value of soil properties. This factor analysis approach was utilized to derive these properties, relying on the ratio of each indicator’s shared variance relative to the total shared variance across all indicators.

The Kaiser–Meyer–Olkin measure, with a value of 0.574, exceeding the threshold of 0.5, and Bartlett’s test of sphericity (*p*-value < 0.001) both supported the appropriateness of using principal component analysis (PCA) [73]. Based on the grading criteria for soil quality, SQI was divided into five distinct categories:

Level I: SQI ≥ 0.85, extremely high;
Level II: 0.85 > SQI ≥ 0.7, high;
Level III: 0.7 > SQI ≥ 0.55, medium;
Level IV: 0.55 > SQI ≥ 0.4, low;
Level V: SQI < 0.4, very low.

### 4.6. Rhizosphere Soil Microbial Community

The Majorbio Company (Shanghai, China) conducted an analysis of microbial communities using freshly prepared rhizosphere soil samples. The total microbial genomic DNA was extracted from these soil samples using the E.Z.N.A.^®^ Soil DNA Kit sourced from Omega Bio-tek in Norcross, GA, USA. The quality and concentration of the extracted DNA were then assessed using both 1.0% agarose gel electrophoresis and a NanoDrop 2000 spectrophotometer manufactured by Thermo Scientific in the United States. Subsequently, the variable regions V3–V4 of the bacterial 16S rRNA gene were amplified, employing the primer pairs 338F (5′-ACTCCTACGGGAGGCAGCAG-3′) and 806R (5′-GGACTACHVGGGTWTCTAAT-3′) through a T100 Thermal Cycler PCR machine (Kapa Biosciences, Woburn, MA, USA) [74]. The taxonomy of each OTU representative sequence was analyzed by RDP Classifier version 2.2 [75] against the 16S rRNA gene database (e.g., Silva v138) using a confidence threshold of 0.7. All raw sequence data were deposited in the National Center for Biotechnology Information (NCBI) platform (Illumina, San Diego, CA, USA); the accession number is PRJN 1173869 (for bacteria) [72].

### 4.7. Statistical Analysis

IBM SPSS Statistics 27 (SPSS, Inc.; Chicago, IL, USA) was employed for data statistics and analysis, with significance calculated at the *p* < 0.05 level through Duncan’s multiple range test. Soil and plant-related indicators were quantitatively analyzed using Origin 2024 (10.1), and a radar chart of SQI was drawn. A correlation analysis between soil environmental factors and microbial communities was performed utilizing the R software (Version 4.4.0). The Gephi software (Version 0.10.1) was used to meticulously create the co-occurrence network diagram to quantify the influence of soil environmental factors on the microbial community. The partial least squares path structural equation model (Smart PLS 4.0 software) was used to identify the primary factors affecting the impact of sludge biochar on plant growth.

## 5. Conclusions

The use of SSB proved effective in boosting the growth of both *ryegrass* and *cosmos*. This method not only improved soil quality but also contributed to the development of the rhizospheric soil microbial composition. The main results were as follows: The addition of 9% and 3% (w w^−1^) SSB achieved the best growth for *ryegrass* and *cosmos*, with aboveground biomass increasing by 68.97% and 68.12%, respectively, and root biomass increasing by 49.87% and 45.14%. SSB improved the soil quality, with the 9% (w w^−1^) SSB addition being optimal for *ryegrass* (SQI > 0.55) and *cosmos* (SQI > 0.7). Researchers using PLS-PM found that the application of SSB significantly impacted soil bacteria. Soil bacteria had significant effects on both pH and EC, and pH negatively affected EC. Environmental factors significantly positively influenced the biomass accumulation and height of *ryegrass* and *cosmos*. Redundancy analysis and Spearman correlation analysis showed that soil nutrient status was a crucial factor influencing the bacterial community structure; contents including SAN, SOM, pH, and SNN were the main factors affecting the structure of bacterial communities. Overall, the application of SSB improved the physicochemical properties and enzyme activities of the soil, positively impacting soil quality and microbial communities. This suggests that the addition of SSB (3–6%, w w^−1^) is an optimal strategy to promote the growth of *ryegrass* and *cosmos*. It provides technical support for the resource utilization of surplus sludge. Currently, most research on the impacts of SSB application on soil and plant growth is based on indoor, short-term, greenhouse cultivation experiments. Therefore, the long-term environmental impacts and mechanisms associated with the addition of sludge biochar require further investigation.

## Figures and Tables

**Table 1:** Rhizosphere soil microbial community α-diversity index.

Treatment	Observed Species	Shannon	Chao1	ACE	Goods Overage	Simpson
*R* *yegrass*						
P1S1	2946	6.436	3296.48	3438.557	0.984	0.005
P1S2	2981	6.187	3339.98	3484.364	0.984	0.016
P1S3	2218	4.193	2477.358	2649.335	0.987	0.181
P1S4	2385	4.856	2675.317	2809.191	0.987	0.125
P1S5	2200	4.877	2406.773	2476.299	0.99	0.114
*Cosmos*						
P2S1	2589	6.358	2853.317	2962.187	0.987	0.006
P2S2	3099	6.571	3391.683	3562.287	0.985	0.007
P2S3	2389	5.223	2705.483	2864.4	0.986	0.073
P2S4	2279	4.968	2516.524	2604.829	0.989	0.096
P2S5	2071	4.604	2294.213	2378.471	0.989	0.125

Note: P1S1, *ryegrass* + 0% SSB, w w^−1^; P1S2, *ryegrass* + 1% SSB, w w^−1^; P1S3, *ryegrass* + 3% SSB, w w^−1^; P1S4, *ryegrass* + 6% SSB, ww^−1^; P1S5, *ryegrass* + 9% SSB, w w^−1^; P2S1, *cosmos* + 0% SSB, w w^−1^; P2S2, *cosmos* + 1% SSB, w w^−1^; P2S3, *cosmos* + 3% SSB, w w^−1^; P2S4, *cosmos* + 6% SSB, w w^−1^; P2S5, *cosmos* + 9% SSB, w w^−1^.

**Table 2:** Topological properties of bacterial and fungal co-occurrence networks under different amendment treatments.

Index		Nodes	Edges	Average Degree	Clustering Coefficient	Betweenness Centralization	Degree Centralization	Negative Edges/%
Group	*Ryegrass*	342	4781	27.959	0.544	0.021	0.082	20.27
	*Cosmos*	382	6611	34.612	0.572	0.022	0.122	32.73

**Table 3:** The R^2^ value and *p* value of redundancy discriminant analysis (RDA) between rhizosphere soil microbial communities and environmental factors.

Envfti Factor	*R* *yegrass*		*C* *osmos*	
	R2	*p* Values	R2	*p* Values
pH	0.3293	0.068	0.7083	0.005 **
CEC	0.176	0.328	0.8384	0.002 **
SOM	0.902	0.001 ***	0.1974	0.269
SAN	0.3622	0.053	0.522	0.015 *
SNN	0.4385	0.013 *	0.7699	0.001 ***
SPEA	0.5273	0.017 *	0.1072	0.501
SCEA	0.3208	0.071	0.1516	0.221
SIEA	0.1543	0.358	0.2874	0.099

Note: Significance levels are denoted as *** for *p* < 0.001 ** for *p* < 0.01 and * for *p* < 0.05.

**Table 4:** Membership values and weights of different indicators are determined for diverse soil treatments based on the fuzzy comprehensive evaluation membership function.

Plant	Treatment	pH	CEC	SOM	SAN	SNN	SPEA	SCEA	SIEA
*Ryegrass*	P1S1	0.787	0.226	0.153	0.308	0.190	0.717	0.532	0.490
	P1S2	0.483	0.101	0.210	0.107	0.566	0.566	0.544	0.574
	P1S3	0.468	0.999	0.553	0.381	0.910	0.526	0.556	0.551
	P1S4	0.313	0.502	0.755	0.993	0.663	0.519	0.568	0.610
	P1S5	0.476	0.816	0.947	0.821	0.313	0.383	0.550	0.526
	Weights	0.263	0.149	0.077	0.136	0.034	0.125	0.062	0.154
*Cosmos*	P2S1	0.271	0.101	0.270	0.101	0.110	0.632	0.417	0.650
	P2S2	0.596	0.714	0.298	0.603	0.990	0.647	0.676	0.462
	P2S3	0.749	0.787	0.161	0.999	0.416	0.613	0.683	0.458
	P2S4	0.812	0.990	0.475	0.446	0.327	0.453	0.432	0.450
	P2S5	0.829	0.999	0.939	0.711	0.246	0.616	0.435	0.460
	Weights	0.254	0.180	0.199	0.103	0.121	0.074	0.042	0.027

**Table 5:** Sewage sludge biochar physicochemical characteristics in the experimental soil.

Sewage Sludge Biochar	Content
pH	8.33 ± 0.56
Electrical conductivity (EC, µS cm^−1^)	389 ± 25.93
Organic matter (SOM, g kg^−1^)	0.53 ± 0.035
Available nitrogen (AN, mg kg^−1^)	93.67 ± 6.25
Available potassium (AK, mg kg^−1^)	8.01 ± 0.53
Total Zn (mg kg^−1^)	240.83 ± 16.06
Total Cu (mg kg^−1^)	188.67 ± 12.58
Total Cr (mg kg^−1^)	512.83 ± 34.19
Total Pb (mg kg^−1^)	19.12 ± 1.267
Total As (mg kg^−1^)	5.24 ± 0.35
Total Cd (mg kg^−1^)	3.12 ± 0.21
Total Ni (mg kg^−1^)	33.17 ± 2.21

## Data Availability

Data are contained within the article.
